# Influence of Conformation of *M*. *tuberculosis* RNase P Protein Subunit on Its Function

**DOI:** 10.1371/journal.pone.0153798

**Published:** 2016-04-18

**Authors:** Alla Singh, Shah Ubaid-ullah, Anup K. Ramteke, Janendra K Batra

**Affiliations:** Immunochemistry Laboratory, National Institute of Immunology, Aruna Asaf Ali Marg, New Delhi -110067, India; Indian Institute of Technology Delhi, INDIA

## Abstract

RNase P is an essential enzyme that processes 5' end leader sequence of pre-tRNA to generate mature tRNA. The bacterial RNase Ps contain a RNA subunit and one protein subunit, where the RNA subunit contains the catalytic activity. The protein subunit which lacks any catalytic activity, relaxes the ionic requirements for holoenzyme reaction and is indispensable for pre-tRNA cleavage *in vivo*. In the current study, we reconstituted the *M*. *tuberculosis* RNase P holoenzyme *in vitro*. We prepared the RNase P protein through two different strategies that differ in the conditions under which the recombinant *M*. *tuberculosis* protein, expressed in *E*. *coli* was purified. The mycobacterial RNase P protein which was purified under native conditions subsequent to isolation from inclusion bodies and *in vitro* renaturation, was capable of cleaving pre-tRNA specifically without the requirement of RNase P RNA. However, the preparation that was purified under denaturing conditions and refolded subsequently lacked any inherent pre-tRNA processing activity and cleaved the substrate only as a component of the holoenzyme with the RNA subunit. We found that the two RNase P protein preparations attained alternative conformations and differed with respect to their stability as well.

## Introduction

In a cell, transfer RNA (tRNA) acts as an adaptor between the coding sequence in messenger RNA and amino acid sequence in proteins [1]. The tRNA must be folded to the correct ‘L-shaped’ tertiary structure in order to participate in protein translation [2]. After transcription, tRNA molecules have extra sequences at 5’ and 3’ ends, referred to as 5’ leader and 3’ trailer, respectively. The tRNA along with its extra sequences is denoted as precursor tRNA (pre-tRNA) [3]. These extra sequences in the pre-tRNA must be removed to form a mature tRNA product that can attain correct conformational shape and take part in the protein synthesis process. This process of removal of extra sequences is referred to as pre-tRNA processing. Several enzymes are involved in this task *in vivo* [4]. The 3’ trailer sequence is processed by enzymes like RNase D, E, F, Z, etc. [5,6]. The 5’ leader sequence is removed by a single endonuclease called RNase P [7].

RNase P is a ribonucleoprotein complex consisting of a catalytic RNA component and one or more protein subunits, depending on the organism [8,9]. The RNA component is the catalytic component of the holoenzyme that contains the active site where phosphodiester bond cleavage takes place to generate mature tRNA product from the pre-tRNA molecule [10]. The RNA component alone can process pre-tRNA, without the RNase P protein, under high ionic concentrations *in vitro* [11]. *In vivo*, the protein component is indispensable for RNase P activity [12]. The bacterial RNase P holoenzyme is composed of an RNA subunit of 330–420 nucleotides and a protein subunit of around 120 amino acids [13]. The archaeal and eukaryotic RNase P holoenzymes consist of one RNA subunit, but the number of protein subunits varies. While in archaea five protein subunits have been recognized, in eukaryotes nine to ten protein subunits have been reported [14,15].

The protein component of RNase P forms a functional complex with the RNA subunit. The protein subunit in RNase P holoenzyme alters the recognition of the pre-tRNA in many ways. This is evident from the fact that the *B*. *subtilis* holoenzyme binds pre-tRNA more tightly than tRNA, whereas the RNA component binds product more tightly [16]. Interactions of RNase P RNA with the 21 to 25 nucleotides long leader sequence and the T stem of pre-tRNA are altered in the presence of the protein component [17], which also lowers the required concentration of magnesium for efficient catalysis [18]. These functional changes in the holoenzyme may be caused by direct contacts between the substrate and the protein and/or by a protein-induced conformational change in the RNA. In *E*. *coli* RNase P, it is shown that the protein subunit's binding site on the RNA component is neither close to the active site nor close to the substrate binding site indicating that the binding of protein might induce some conformational changes in RNA that lead to the enhanced activity of holoenzyme compared to RNA alone [19]. In *B*. *subtilis* RNase P, although no cross-linking contacts are observed between protein and the mature sequence of pre-tRNA, RNase P protein contacts the single-stranded leader sequence of pre-tRNA [20]. The cross-linking data confirm that the RNase P protein is involved in substrate binding. The protein components of various bacterial RNase Ps have two conserved motifs, namely the RNR motif and Central Cleft, which are respectively involved in protein's interaction with the RNA component and substrate [13,16].

Like in other bacteria, *M*. *tuberculosis* RNase P is also composed of one RNA and one protein subunit. The RNR motif and central cleft are generally conserved in the mycobacterial enzymes, though it also contains few unique residues in these regions. Earlier, we have functionally characterized the RNase P enzyme of *M*. *tuberculosis* [21]. The protein and RNA components of *M*. *tuberculosis* RNase P were produced in *E*. *coli in vitro* and reconstituted to generate a functionally active enzyme [21]. During our earlier study it was observed that the protein component of *M*. *tuberculosis* RNase P, made under certain conditions, was able to process pre-tRNA independently. In this study, the mycobacterial RNase P protein was recombinantly produced in *E*. *coli* by two different strategies. One of the two protein preparations manifested pre-tRNA processing activity in the absence of RNA component, which is unusual for a bacterial RNase P. The study demonstrates that the RNase P protein component of *M*. *tuberculosis* is capable of attaining a conformation which imparts catalytic activity to the protein.

## Materials and Methods

### Expression and Purification of *M*. *tuberculosis* RNase Protein Component P in *E*. *coli*

The gene encoding RNase P protein component is annotated as rnpA in *M*. *tuberculosis* genome. The DNA encoding the protein component of mycobacterial RNase P was cloned and expressed in *E*. *coli* as described earlier [21]. The *E*. *coli* strain BL21 (λDE3) (New England Biolabs, USA) was transformed with the expression vector pVex11 containing the rnpA gene. The culture was grown in superbroth medium containing 0.1 mg/ml ampicillin at 37°C with shaking. The culture was induced with 1 mM IPTG at A600 of 1–1.2. The cells were harvested 2 hours after induction by centrifugation at 4000xg at 4°C for 15 minutes. The recombinant protein was localized in the inclusion bodies.

Two different protocols were subsequently followed to denature, renature and purify the recombinant protein from the inclusion bodies. In the first protocol, which was used in our earlier study [21], the purified inclusion bodies were dissolved in Buffer A that had 8 M urea in 20 mM Tris-Cl, pH 8. The denatured protein was loaded on a SP-sepharose, cation exchange column equilibrated with Buffer A. Extensive washing of the column was done with 10 column volumes. Elution of the bound protein was done using a gradient 0 to 2 M sodium chloride in Buffer A. The fractions collected were run analyzed by SDS-PAGE and those containing the RNase P protein were pooled. Step-wise dialysis of the pooled protein was performed in order to gradually remove urea completely and renature the protein. The protein was finally brought in 20 mM Tris-Cl, pH 8. This protein preparation is denoted as ''RNase P-U protein'' in the manuscript.

In the second protocol, the inclusion bodies were dissolved in Buffer B containing 6M guanidine-hydrochloride in 1M Tris-Cl, 0.5 M EDTA, pH 8. The denatured protein was oxidized using 0.9 mM glutathione and kept for renaturation by 100-fold dilution in Buffer C containing 0.1 M Tris-Cl, pH 8, 0.5 M L-arginine and 2 mM EDTA, for 36 hours. The renatured protein was dialyzed against 0.1 M urea in MES buffer, pH 6. The protein was then subjected to cation-exchange chromatography on a SP sepharose column. The bound protein was eluted using a gradient of 0 to 2 M sodium chloride in MES buffer, pH 6. The fractions were analyzed by SDS-PAGE and those containing the RNase P protein were pooled. The protein was subjected to size-exclusion chromatography on Superose 12 column in Phosphate buffered saline, pH 7.4. The fractions containing the RNase P protein were collected. This protein preparation is denoted as ''RNase P-G'' in the manuscript.

N-terminal sequencing confirmed the authenticity of the two protein preparations. The concentration of the purified protein was estimated by Bradford's method [22]. SDS-PAGE was performed by Laemmli’s method [23] to analyze the purity of proteins.

### CD Spectral Analysis of Proteins

Far-UV (250–200 nm) and Near-UV (320–250 nm) CD spectra were measured using a cell of 0.1 cm and 1 cm path-length, respectively in JASCO spectropolarimeter (Model- J815) attached to a peltier temperature controller. The spectra were recorded at a scan rate of 100 nm.min-1. For each spectrum, at least 10 scans were taken. Protein concentration of 12 μM and RNA concentration of 2 μM was used.

### Cloning of DNAs Encoding RNA Component of RNase P and Precursor tRNA Alanine of *M*. *tuberculosis*

The DNAs encoding RNA component of mycobacterial RNase P and an isoform of alanine tRNA, annotated as AlaU, were cloned in *E*. *coli* vectors as described earlier [21]. *In vitro* transcription was performed, using a modified protocol of Kreig and Melton [24], to make RNase P RNA component and the pre-tRNA substrate [21].

### Assay for Enzymatic Activity of RNase P

The reaction to assay the pre-tRNA processing contained 50 mM Tris-Cl pH-7.4, 500 mM ammonium acetate, 10 mM magnesium chloride for RNase P-G holoenzyme, and 50 mM Tris-Cl pH-7.4, 100 mM ammonium acetate, 10 mM magnesium chloride for RNase P-U holoenzyme with indicated concentrations of RNase P RNA and/or protein and labeled substrate pre-tRNA_ala_ in 20 μl reaction volume. In case of reactions containing RNase P RNA, it was pre-incubated at 50°C for 30 minutes, followed by cooling to room temperature, and then the protein component was added. The substrate pre-tRNA_ala_ was added last. The reaction was kept at 37°C for 10 minutes. A 10% acrylamide gel containing 7 M Urea was used to separate the pre-tRNA substrate and the mature product. The contents of the gel were visualized by autoradiography. The bands on the autoradiogram were quantified densitometrically on an Alpha Imager gel documentation system. The data were processed to obtain relative product formation.

### Zymogram Analysis of Proteins

A zymogram analysis was performed as described by Blank et al. [25] to analyze RNase activity in various proteins. Proteins were run on 15% SDS-polyacrylamide gel containing 0.3 mg/ml yeast tRNA under non-reducing conditions. After the electrophoresis, SDS was removed by washing in 25% isopropanol. Gels were incubated at room temperature for 15 minutes and stained for undigested RNA with 0.2% toluidine blue. Appearance of clear zones against a blue background indicates ribonucleolytic activity.

### Micrococcal Nuclease and Proteinase K Treatment

A typical reaction mixture to treat RNase P components with micrococcal nuclease (MN) contained 50 mM Tris-HCl buffer, pH 9, 2 mM calcium chloride and 300 U/ml MN as suggested by the manufacturer (Sigma Chemical Company). The reaction was incubated at 37°C for 30 minutes and stopped by addition of 10 mM ethylene glycol tetraacetic acid (EGTA).

The RNase P components were treated with 25 mg/ml Proteinase K (PK) in 50 mM Tris-HCl buffer, pH 8 and 1.5 mM calcium chloride at 37°C for 30 minutes.

After the treatment with nuclease or proteinase, the treated components were assayed for pre-tRNA processing activity using standard conditions of 10 mM magnesium chloride, 100 mM ammonium acetate in 50 mM Tris-HCL pH 7.4 for RNase P-G and RNase P-U proteins; and 500 mM magnesium chloride, 500 mM ammonium acetate in 50 mM Tris-HCl pH 7.4 for RNase P RNA alone, at 37°C for 10 minutes.

### Statistical Analysis

Mean and Standard Error, shown in the manuscript have been calculated using MS Excel.

## Results

### Expression of the Components of the RNase P of *M*. *tuberculosis*

**Fig 1:**
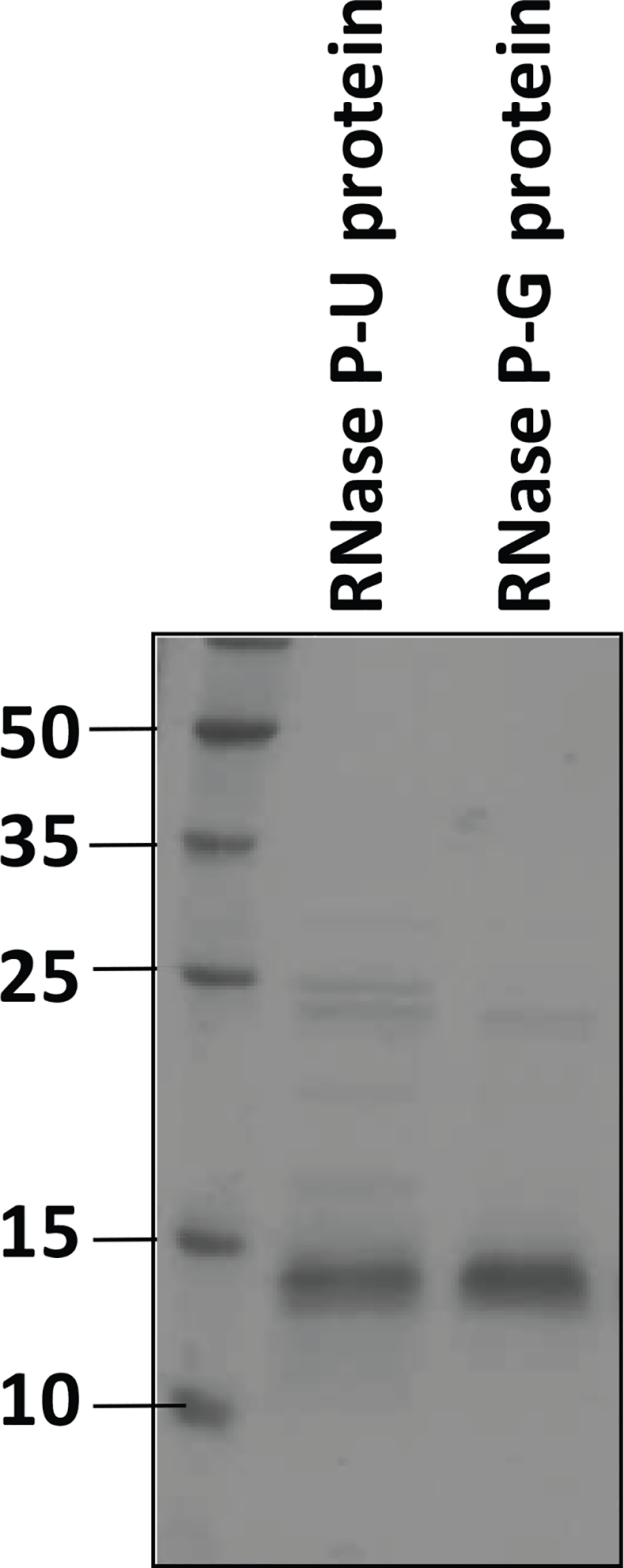
SDS-PAGE analysis of purified protein preparations. The two purified protein preparations of *M*. *tuberculosis* RNase P expressed in *E*. *coli* were analysed by SDS-polyacrylamide gel electrophoresis on a 14% gel. Molecular weight markers are shown in kDa.

The DNA encoding RNase P protein component, cloned in expression vector pVex11, was expressed in *E*. *coli* BL21 (λDE3) cells. The expressed protein was localized in the inclusion bodies. The protein component of RNase P of *M*. *tuberculosis* was further purified from inclusion bodies by two different methods resulting in two protein preparations termed RNase P-U and RNase P-G protein, respectively (Fig 1). The holoenzymes obtained by reconstitution of two proteins with RNase P RNA are termed as RNase P-U holoenzyme and RNase P-G holoenzyme.

### Characterization of Pre-tRNA Cleavage Activity of *M*. *tuberculosis* RNase P

**Fig 2:**
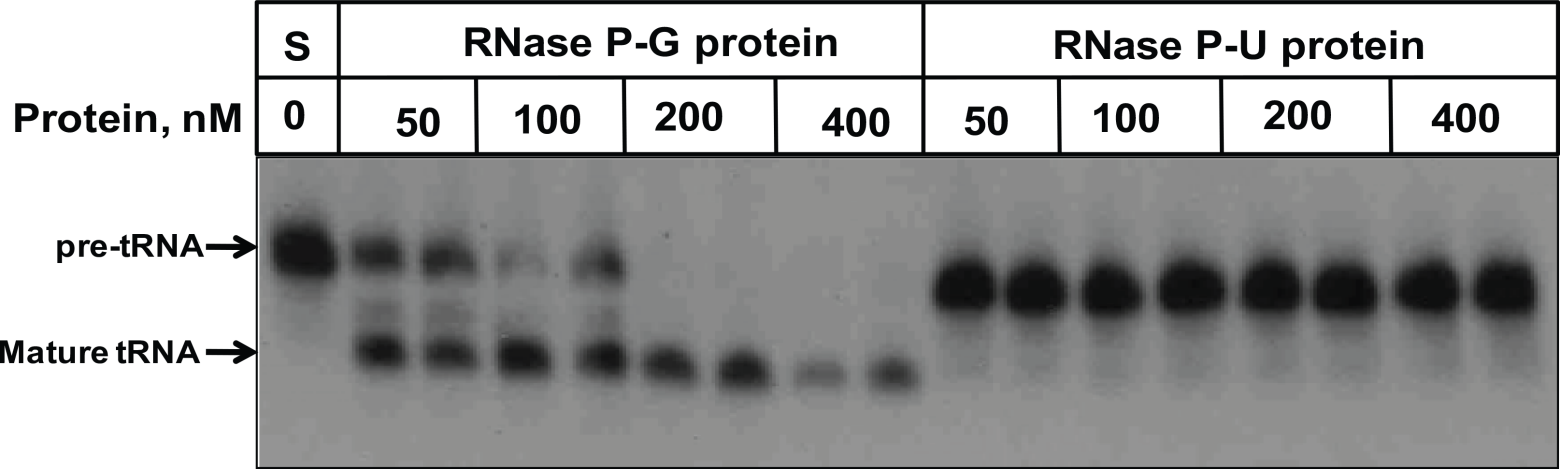
Activity of protein preparations on pre-tRNA at low ammonium acetate. The pre-tRNA processing activity with different amounts of RNase P protein preparations alone, in the absence of RNA component, was assayed in 50 mM Tris-HCl (pH 7.4), 10 mM magnesium chloride and 100 mM ammonium acetate. S denotes the substrate alone reaction.

The two preparations of the RNase P protein component were analyzed for their effect on the pre-tRNA substrate (Fig 2). The RNase P-G protein was found to cleave the pre-tRNA by itself in a dose dependent manner, whereas the RNase P-U protein did not cleave pre-tRNA at all (Fig 2). RNase P-G protein showed complete processing of pre-tRNA beyond 200 nM concentration of the protein (Fig 2).

### Comparison of Pre-tRNA Processing Activities of RNase P-G Holoenzyme and RNase P-U Holoenzyme

**Fig 3:**
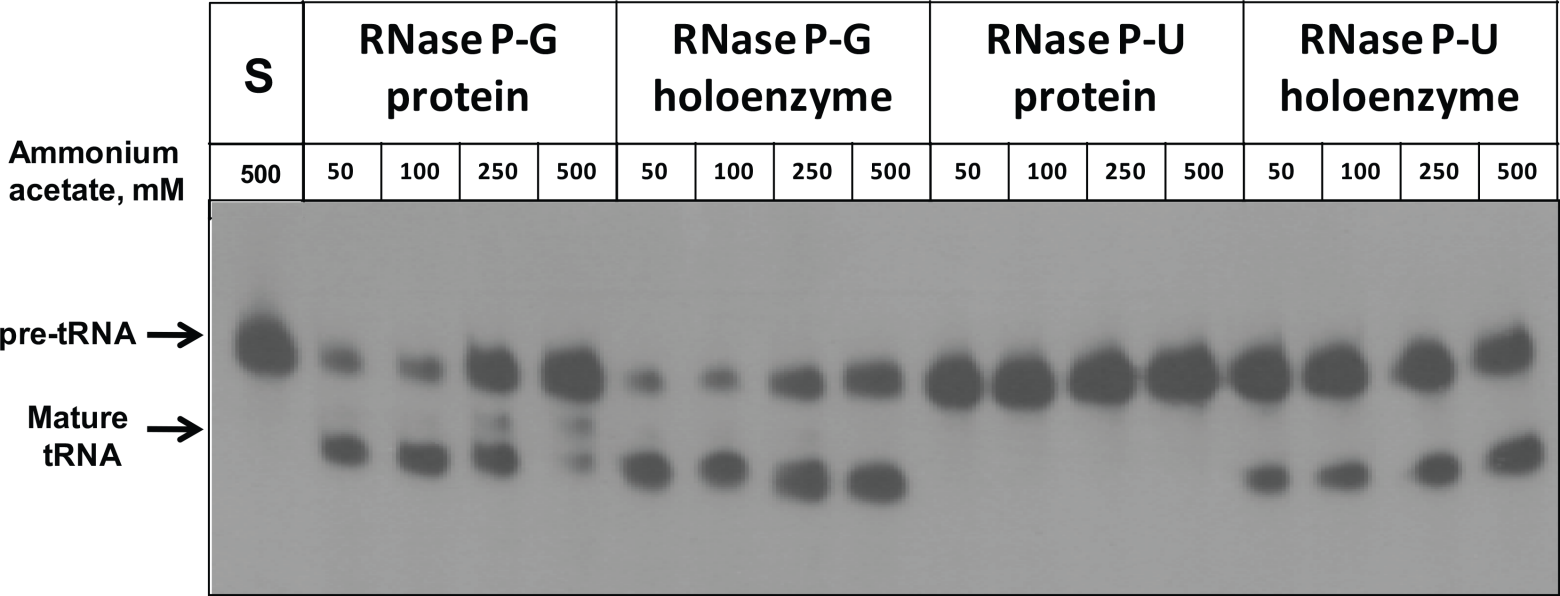
Activity of RNase P-G and RNase P-U holoenzyme complexes at different ammonium acetate concentrations. The pre-tRNA processing activity of RNase P-G and RNase P-U proteins and their holoenzymes was assayed in the presence of different concentrations of ammonium acetate. To reconstitute the RNase P holoenzyme, 50 nM RNase P RNA and 100 nM RNase P protein were used. For protein alone activity, 100 nM protein was used in the assays.

The activity of the holoenzyme complexes reconstituted separately with RNA component and RNase P-U and RNase P-G protein components was checked on pre-tRNA substrate at various concentrations of ammonium acetate (Fig 3). Both holoenzymes showed pre-tRNA processing activity with increasing ammonium acetate concentration (Table 1). The activity of RNase P-G protein alone was inhibited with increasing concentration of ammonium acetate and it had negligible activity at 500 mM ammonium acetate (Fig 3, Table 1). The RNase P-U protein by itself did not show any pre-tRNA cleavage at any ammonium acetate concentration (Fig 3).

**Table 1:** Catalytic activity of *M tuberculosis* RNase P protein preparations and holoenzymes.

Ammonium acetate, mM	RNase P-G holoenzyme*	RNase P-G protein*	RNase P-G holoenzyme*	RNase P-G holoenzyme**	RNase P-U holoenzyme*	RNase P-U protein*	RNase P-U holoenzyme*	RNase P-U holoenzyme**
50	61 ± 41	53 ± 30	8 ± 14	66 ± 12	25 ± 18	0	25 ± 18	21 ± 15
100	63 ± 59	60 ± 52	3 ± 15	25 ± 13	30 ± 33	0	30 ± 33	25 ± 27
250	53 ± 53	42 ± 70	11 ± 25	86 ± 20	35 ± 39	0	35 ± 39	29 ± 32
500	43 ± 39	13 ± 20	30 ± 19	245 ± 16	46 ± 48	0	46 ± 48	38 ± 40

The % product formed in case of RNase P-G and RNase P-U protein alone, and holoenzymes has been shown. The holoenzyme activity has been calculated by subtracting the % product formed by protein alone from that of the holoenzyme. The holoenzyme activity has been further converted to μM product/mM enzyme/second. The activities shown are *% product formed or **μM product/mM enzyme/second. The enzyme activities are Mean ± SE of three independent values.

The activity of holoenzyme complex reconstituted with RNase P-G protein was calculated by subtracting the protein-alone activity from that of the holoenzyme activity for the respective proteins (Table 1). The RNase P-U holoenzyme showed higher pre-tRNA processing than RNase P-G holoenzyme at all concentrations of ammonium acetate (Table 1).

### Comparison of Pre-tRNA Processing by RNase P RNA and RNase P-G Protein and Zymogram Analysis of Proteins

**Fig 4:**
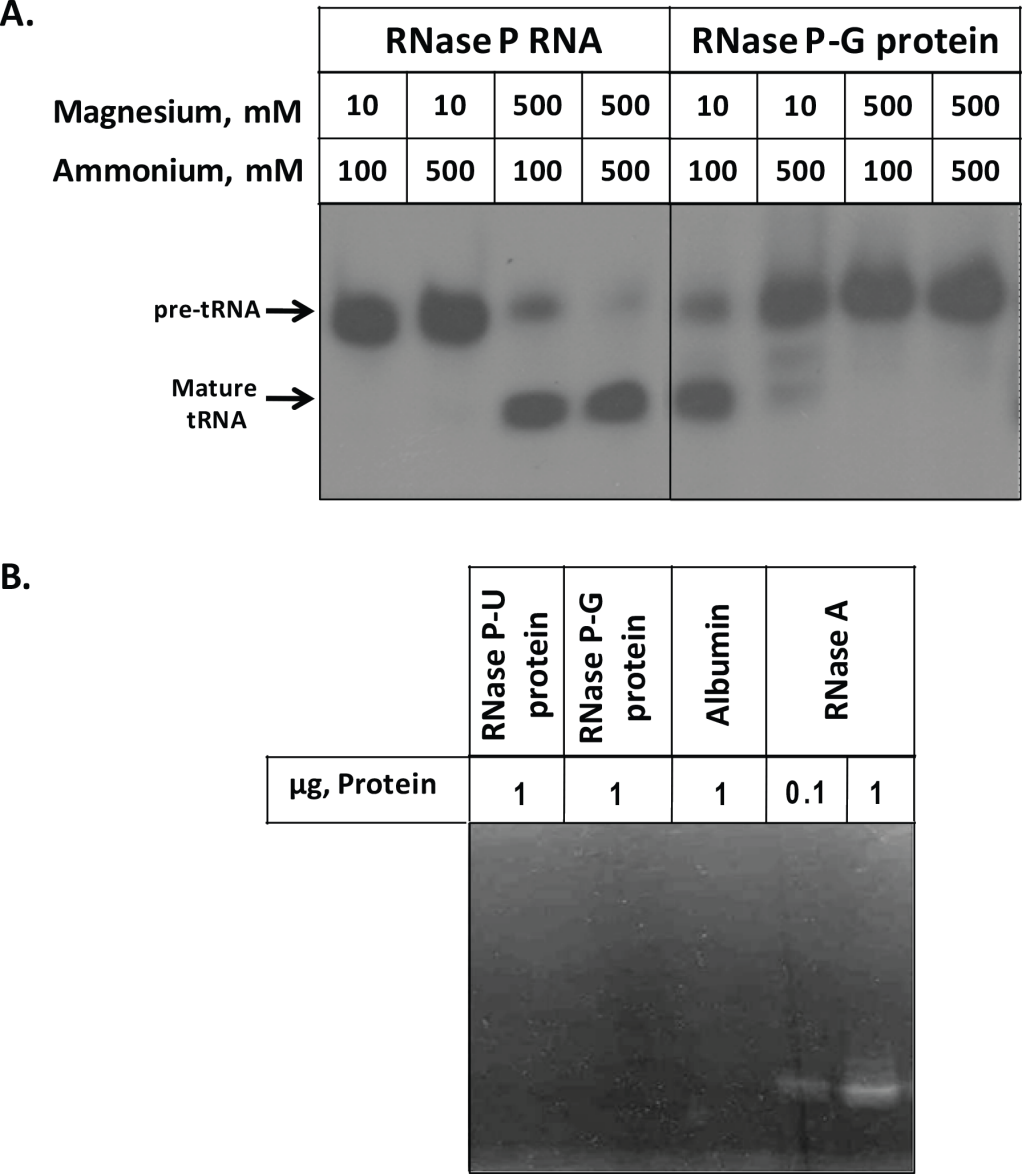
Comparison of pre-tRNA processing by RNase P RNA and RNase P-G protein and zymogram analysis of protein preparations. **A.** Indicated concentrations of magnesium and ammonium ions were used to assay the pre-tRNA cleavage by RNase P RNA and RNase P-G protein. The reactions contained 50 nM of RNase P RNA and 100 nM of RNase P-G protein. **B.** Zymogram analysis of the two protein preparations, albumin and RNase A.

One of the reasons for catalytic activity being manifested by RNase P-G protein could be the presence of RNA component of *E*. *coli* RNase P which may be co-purifying with the protein. To investigate the possibility of any contaminating RNA in the RNase P-G protein preparation, different combinations of magnesium and ammonium ions were used to assess the pre-tRNA processing by RNase P RNA and RNase P-G protein (Fig 4A). As shown in Fig 4A, RNase P RNA cleaves the pre-tRNA *in vitro* at high concentrations of magnesium and ammonium ions. On the other hand, RNase P-G protein processed pre-tRNA only at low concentrations of magnesium and ammonium ions, suggesting it to be an activity of the protein (Fig 4A).

A zymogram analysis of the two protein preparations was done using yeast-tRNA as the substrate to analyze if the RNase P-G protein had a contaminating RNase or the protein itself contained non-specific ribonucleolytic activity. The two RNase P protein preparations and the BSA, taken as negative control did not show any cleavage of yeast tRNA (Fig 4B). RNase A, used as positive control showed efficient digestion of yeast tRNA at as low as 0.1 μg amount (Fig 4B).

### Treatment of Components of RNase P with Nuclease and Proteinase K

**Fig 5:**
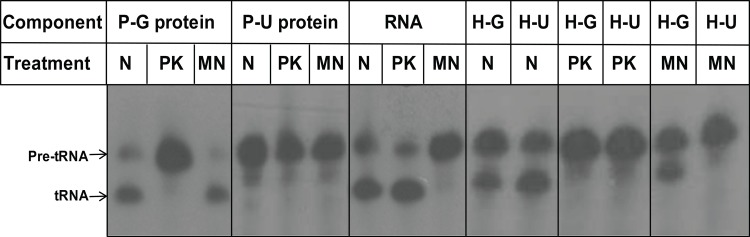
Pre-tRNA processing by components of RNase P upon treatment with micrococcal nuclease and proteinase K. RNase P-G protein, RNase P-U protein and RNase P RNA were treated with either micrococcal nuclease (MN) or proteinase K (PK), followed by activity assay using standard conditions. N, untreated sample; MN, treated with micrococcal nuclease; PK, treated with proteinase K; H-G and H-U refer to holoenzymes reconstituted with RNase P-G and RNase P-U proteins, respectively.

The RNase P components, RNase P-G and RNase P-U proteins, and RNase P RNA were separately treated with micrococcal nuclease to determine if the protein preparation contained any contaminating RNA component of *E*. *coli*. After the treatment, micrococcal nuclease was inactivated by addition of EGTA to the reaction as it requires calcium ions for activity. The components were separately treated with proteinase K also to confirm that the activity seen with RNase P-G protein was within the protein. After treatment with nuclease and proteinase, the treated components were used to assay the pre-tRNA processing activity using standard conditions. The activity of RNase P-G protein alone was abolished upon treatment with proteinase K, however it was unaffected after treatment with micrococcal nuclease (Fig 5). As seen before, RNase P-U protein did not show any activity by itself under any conditions (Fig 5). The RNase P RNA alone activity was abolished upon treatment with micrococcal nuclease, and remained unaffected after treatment with proteinase K (Fig 5). When the holoenzymes, reconstituted with RNA component and RNase P-G and PU proteins were treated with proteinase K, their activities were lost (Fig 5). However, treatment of holoenzymes with nuclease resulted in the loss activity of that with P-U protein, whereas there was activity in RNase P-G holoenzyme confirming it to be the activity of RNase P-G protein alone (Fig 5).

### Effect of Temperature on the Activity of RNase P Protein Preparations

**Fig 6:**
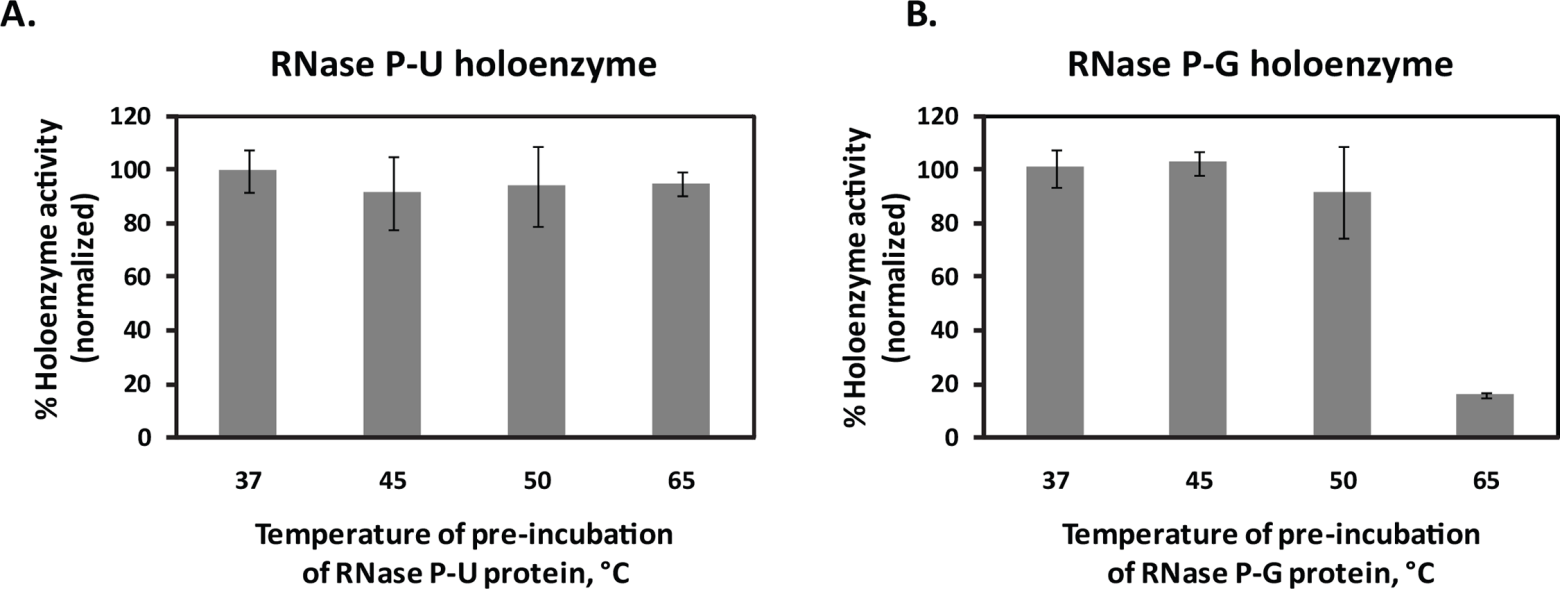
Effect of temperature on the activity of the protein preparations. The two protein preparations, 400 nM each were pre-incubated at indicated temperatures and further used to reconstitute holoenzymes with the 200 nM RNA component. **A.** RNase P-U holoenzyme. **B.** RNase P-G holoenzyme. The % product formation in each case was normalized with respect to that at 37°C, which is shown as 100%. Data are plotted as Mean ± SD of three independent observations.

In order to assess the stability of the two RNase P proteins, they were pre-incubated at 37°, 45°, 50° and 65°C for 30 minutes and subsequently, reconstituted with RNase P RNA to form holoenzymes. While the RNase P-U holoenzyme retained similar pre-tRNA processing activity even after the protein being exposed to high temperatures (Fig 6A), RNase P-G holoenzyme lost considerable activity at 65°C compared to that at 37°C (Fig 6B). This shows that the two protein preparations differ with respect to their stability at higher temperatures.

### CD Spectroscopy of the *M*. *tuberculosis* RNase P Protein Preparations

**Fig 7:**
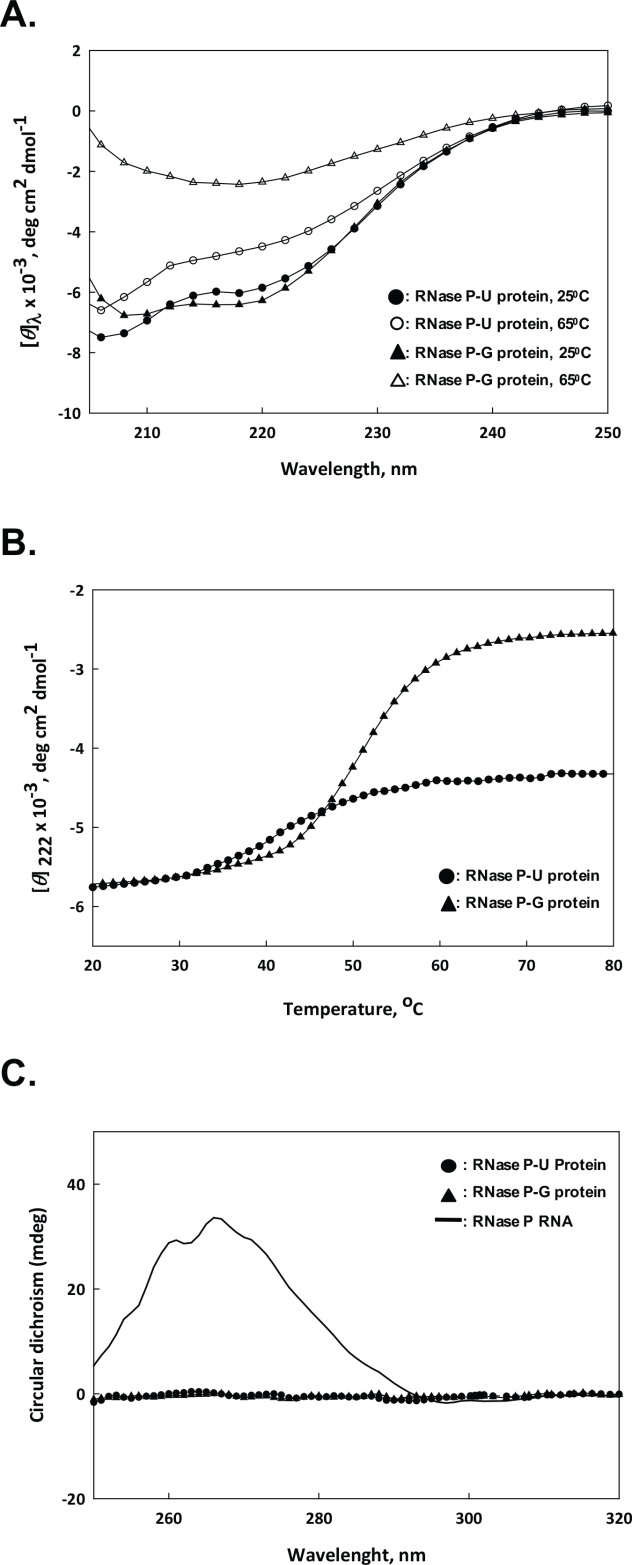
CD spectroscopy of RNase P protein components. **A.** Far-UV CD spectra of the two protein preparations from 205–250 nm at different temperatures. **B.** CD profile of RNase P-U protein and RNase P-G protein within the temperature range of 20–80°C. **C.** Near-UV CD spectra of RNase P RNA (2 μM), and RNase P-U protein and RNase P-G protein (12 μM each) within 250–320 nm.

CD spectroscopy of the two protein preparations was done at 25°C and 65°C (Fig 7A). Both the proteins showed similar structure at 25°C (Fig 7A). However, when exposed to 65°C, RNase P-G protein lost considerable secondary structure compared to that at 25°C, whereas RNase P-U protein did not lose its secondary structure significantly at 65°C (Fig 7A). At 65°C, the CD spectra for both the proteins showed noise beyond 205 nm, so scans have been shown for the 205–250 nm region.

Further, we checked the loss of structure with increase in temperature by monitoring change in [*θ*]222 versus temperature within 20°C to 80°C (Fig 7B). RNase P-G protein lost its secondary structure significantly, whereas RNase P-U protein was not completely denatured even at 80°C (Fig 7B).

To further establish no RNA contamination in RNase P-G protein preparation, near UV-CD spectra of RNase P RNA, and RNase P-U protein and RNase P-G protein were recorded (Fig 7C). RNA showed a typical peak at 265 nm, whereas a much higher concentration of the two proteins did not show any peak in the near UV region (Fig 7C).

## Discussion

RNase P has been shown to be essential for survival of *M*. *tuberculosis* [26]. The need for new drug targets to counter the evolution of drug-resistance in mycobacterium has emphasized the need for characterization of molecules essential for the survival of *M*. *tuberculosis* [27,28]. The detailed characterization of RNase P enzyme of *M*. *tuberculosis* described in the current study has implications in exploring this enzyme as a drug target.

The *M*. *tuberculosis* RNase P protein component was over-expressed in *E*. *coli* where the protein accumulated in inclusion bodies. The protein was purified from inclusion bodies via two protocols that differed in the denaturing agent used to solubilize inclusion bodies and the conditions under which the protein was purified. While RNase P-G protein was solubilized in guanidine, renatured *in vitro* and purified under native conditions, the RNase P-U protein was solubilized in urea and purified under denaturing conditions, followed by step-wise dialysis to renature the protein. The RNase P-U protein did not show any pre-tRNA processing activity as expected of bacterial RNase P proteins, and was used in our earlier study [21]. However, it was surprising to note that RNase P-G protein cleaved pre-tRNA at low ammonium acetate concentration without the requirement of RNase P RNA. The pre-tRNA processing activities of RNase P-G and RNase P-U holoenzymes, obtained by reconstitution with RNase P RNA, significantly differed under different ionic conditions. The RNase P-G holoenzyme cleaved pre-tRNA in the presence of ammonium acetate upto 500 mM, while the activity of RNase P-G protein was almost completely inhibited at 500 mM ammonium acetate. RNase P-U holoenzyme cleaved pre-tRNA more efficiently than RNase P-G holoenzyme at all concentrations of ammonium acetate used.

It is well known that all RNase P RNAs process pre-tRNA efficiently at high ionic concentrations *in vitro* [29]. RNase P-G protein cleaved pre-tRNA only at low concentrations of magnesium and ammonium ions, whereas RNase P RNA cleaved pre-tRNA at higher concentrations of magnesium. The differential pre-tRNA processing behaviors by RNase P RNA and RNase P-G protein counters the possibility of an RNA contamination in the protein preparation acquired from the host during the purification. The possibility of RNA contamination was further ruled out by CD spectral analysis. It is also noteworthy that during size-exclusion chromatography, the RNase P-G protein elutes at the size corresponding to 14 kDa as confirmed by molecular weight markers (data not shown), whereas any contaminating co-purifying RNA would have caused RNase P-G protein to elute much earlier owing to bigger mass. Further, the pre-tRNA processing activity of RNase P-G protein was abolished upon its treatment with proteinase K, whereas it was not affected by treatment with micrococcal nuclease confirming that the activity seen is associated with the protein and not due to the presence of any contaminating RNA. To investigate the possibility of any contaminating ribonucleolytic protein in RNase P-G protein preparation, zymogram analysis with yeast tRNA as the substrate was done. Neither RNase P-G protein nor RNase P-U protein cleaved yeast tRNA by themselves or showed any contaminating RNase in the preparation. Moreover, it is evident from the study that the pre-tRNA substrate is cleaved specifically by the P-G protein and not completely digested, as would have been the case with a non-specific ribonuclease contamination. The two protein preparations, as a component of the holoenzyme, differ with respect to their pre-tRNA processing activities at different temperatures, with RNase P-U protein being more thermostable. CD spectroscopy analysis further confirmed that while RNase P-U protein retained much of its secondary structure at 65°C, RNase P-G protein was nearly denatured at 65°C. The CD profiles of the two proteins taken in a temperature range of 20°C to 80°C also showed the loss of structure in RNase P-G protein upon increase in temperature. The loss of structure in case of RNase P-U protein was much less than that of RNase P-G protein. Hence, it is clear that RNase P-U and RNase P-G proteins exist in different conformations which govern their respective properties.

Although catalytic activity has been demonstrated for RNase P RNA of some archaea [30], it has long been thought that the eukaryotic RNase P RNA is inactive without the protein components [31]. However, the RNase P RNA of lower eukaryote *Giardia lamblia* and human have been shown to be catalytically active without protein subunits also [32]. Earlier studies with human mitochondria and spinach chloroplasts were suggestive of a type of RNase P that does not contain RNA [33,34]. Detailed characterization of human mitochondria and plant organelles has led to the discovery of single proteins denoted as PRORP (PROteinaceous RNase P) that function without an RNA component [35–37]. Various molecular mechanisms, including compartmentalization of intracellular milieu in case of endosymbiosis and genome compaction, have been suggested to be responsible for the evolution of proteinaceous RNase P [38]. Both, the RNA-based and protein-based RNase P facilitate the cleavage at putative phosphodiester bond by bringing metal ions in its vicinity [39,40].

*M*. *tuberculosis* has a lifestyle of both a host-restricted symbiont and a pathogen [41]. When *M*. *tuberculosis* becomes dormant, protein synthesis is shut down. These dormant mycobacteria restart the protein synthesis machinery when activated by external stimuli, that include oxygen and/or heat shock [42]. Hence, the protein synthesis is required for both actively growing mycobacteria and the dormant bacteria that are undergoing reactivation. It is not clear if the observations made in the current study are seen only *in vitro* and are non-physiological. However, there is a possibility that under specific circumstances an alternative, catalytically competent, conformation of RNase P protein component of *M*. *tuberculosis* gives the pathogen a survival advantage. Greater flexibility at the level of protein synthesis would likely be an advantage to the pathogen for adaptation.

It is interesting to note that mycobacterial RNase P protein can attain a conformation capable of cleaving the pre-tRNA specifically. The capabilities of *M*. *tuberculosis* RNase P, to work as an RNA-based as well as a protein-based enzyme under different ionic conditions, is a novel functionality that warrants further investigation.

## References

[1] Rodnina MV, Wintermeyer W (2011) The ribosome as a molecular machine: the mechanism of tRNA-mRNA movement in translocation. Biochem Soc Trans 39:658–662. 10.1042/BST0390658 21428957

[2] Demeshkina N, Jenner L, Yusupova G, Yusupov M (2010) Interactions of the ribosome with mRNA and tRNA. Curr Opin Struct Biol 20:325–332. 10.1016/j.sbi.2010.03.002 20392630

[3] Bernhardt D, Darnell JE Jr (1969) tRNA synthesis in HeLa cells: a precursor to tRNA and the effects of methionine starvation on tRNA synthesis. J Mol Biol 42:43–56. 4185508 10.1016/0022-2836(69)90485-9

[4] O'Connor JP, Peebles CL (1991) In vivo pre-tRNA processing in *Saccharomyces cerevisiae*. Mol Cell Biol 11:425–439. 1986237 10.1128/mcb.11.1.425-439.1991 PMC359644

[5] Maraia RJ, Lamichhane TN (2011) 3' processing of eukaryotic precursor tRNAs. Wiley Interdiscip Rev RNA 2:362–375. 10.1002/wrna.64 21572561 PMC3092161

[6] Morl M, Marchfelder A (2001) The final cut The importance of tRNA 3'-processing. EMBO Rep 2:17–20. 11252717 10.1093/embo-reports/kve006 PMC1083803

[7] Kirsebom LA (2007) RNase P RNA mediated cleavage: substrate recognition and catalysis. Biochimie 89:1183–1194. 17624654 10.1016/j.biochi.2007.05.009

[8] Evans D, Marquez SM, Pace NR (2006) RNase P: interface of the RNA and protein worlds. Trends Biochem Sci 31:333–341. 16679018 10.1016/j.tibs.2006.04.007

[9] Esakova O, Krasilnikov AS (2010) Of proteins and RNA: the RNase P/MRP family. RNA 16:1725–1747. 10.1261/rna.2214510 20627997 PMC2924533

[10] Reiter NJ, Osterman A, Torres-Larios A, Swinger KK, Pan T, Mondragon A (2010) Structure of a bacterial ribonuclease P holoenzyme in complex with tRNA. Nature 468:784–789. 10.1038/nature09516 21076397 PMC3058908

[11] Guerrier-Takada C, Gardiner K, Marsh T, Pace N, Altman S (1983) The RNA moiety of ribonuclease P is the catalytic subunit of the enzyme. Cell 35:849–857. 6197186 10.1016/0092-8674(83)90117-4

[12] Turrini PC, Loveland JL, Dorit RL (2012) By any other name: heterologous replacement of the *Escherichia coli* RNase P protein subunit has *in vivo* fitness consequences. PLoS One 7:e32456 10.1371/journal.pone.0032456 22448220 PMC3308948

[13] Kazantsev AV, Pace NR (2006) Bacterial RNase P: a new view of an ancient enzyme. Nat Rev Microbiol 4:729–740. 16980936 10.1038/nrmicro1491

[14] Hall TA, Brown JW (2002) Archaeal RNase P has multiple protein subunits homologous to eukaryotic nuclear RNase P proteins. RNA 8:296–306. 12003490 10.1017/s1355838202028492 PMC1370252

[15] Xiao S, Scott F, Fierke CA, Engelke DR (2002) Eukaryotic ribonuclease P: a plurality of ribonucleoprotein enzymes. Annu Rev Biochem 71:165–189. 12045094 10.1146/annurev.biochem.71.110601.135352 PMC3759807

[16] Kurz JC, Niranjanakumari S, Fierke CA (1998) Protein component of *Bacillus subtilis* RNase P specifically enhances the affinity for precursor-tRNAAsp. Biochemistry. 37:2393–2400. 9485387 10.1021/bi972530m

[17] Crary SM, Niranjanakumari S, Fierke CA (1998) The protein component of *Bacillus subtilis* ribonuclease P increases catalytic efficiency by enhancing interactions with the 5' leader sequence of pre-tRNAAsp. Biochemistry 37:9409–9416. 9649323 10.1021/bi980613c

[18] Kurz JC, Fierke CA (2002) The affinity of magnesium binding sites in the *Bacillus subtilis* RNase P x pre-tRNA complex is enhanced by the protein subunit. Biochemistry 41:9545–9558. 12135377 10.1021/bi025553w

[19] Westhof E, Wesolowski D, Altman S (1996) Mapping in three dimensions of regions in a catalytic RNA protected from attack by an Fe(II)-EDTA reagent. J Mol Biol 258:600–613. 8636995 10.1006/jmbi.1996.0272

[20] Niranjanakumari S, Stams T, Crary SM, Christianson DW, Fierke CA (1998) Protein component of the ribozyme ribonuclease P alters substrate recognition by directly contacting precursor tRNA. Proc Natl Acad Sci U S A 95:15212–15217. 9860948 10.1073/pnas.95.26.15212 PMC28022

[21] Singh A, Ramteke AK, Afroz T, Batra JK (2016) Insight into the role of histidine in RNR motif of protein component of RNase P of *M*. *tuberculosis* in catalysis. IUBMB Life 1 24 10.1002/iub.1472 26804985

[22] Bradford M M (1976) A rapid and sensitive method for the quantitation of microgram quantities of protein utilizing the principle of protein-dye binding. Anal Biochem 72:248–254. 942051 10.1016/0003-2697(76)90527-3

[23] Laemmli UK (1970) Cleavage of structural proteins during the assembly of the head of bacteriophage T4. Nature 227:680–685. 5432063 10.1038/227680a0

[24] Krieg PA, Melton DA (1984) Functional messenger RNAs are produced by SP6 in vitro transcription of cloned cDNAs. Nucleic Acids Res 12:7057–7070. 6207484 10.1093/nar/12.18.7057 PMC320142

[25] Blank A, Sugiyama RH, Dekker CA (1982) Activity staining of nucleolytic enzymes after sodium dodecyl sulfate-polyacrylamide gel electrophoresis: use of aqueous isopropanol to remove detergent from gels. Anal Biochem 120:267–275. 6178316 10.1016/0003-2697(82)90347-5

[26] Zhang YJ, Ioerger TR, Huttenhower C, Long JE, Sassetti CM, Sacchettini JC, Rubin EJ (2012) Global assessment of genomic regions required for growth in *Mycobacterium tuberculosis*. PLoS Pathog 8:e1002946 10.1371/journal.ppat.1002946 23028335 PMC3460630

[27] O'Brien RJ, Nunn PP (2001) The need for new drugs against tuberculosis: Obstacles, opportunities, and next steps. Am J Respir Crit Care Med 163:1055–1058. 11316634 10.1164/ajrccm.163.5.2007122

[28] Koul A, Arnoult E, Lounis N, Guillemont J, Andries K (2011) The challenge of new drug discovery for tuberculosis. Nature 469:483–490. 10.1038/nature09657 21270886

[29] Gossringer M, Helmecke D, Hartmann RK (2012) Characterization of RNase P RNA activity. Methods Mol Biol 848:61–72. 10.1007/978-1-61779-545-9_5 22315063

[30] Pannucci JA, Haas ES, Hall TA, Harris JK, Brown JW (1999) RNase P RNAs from some Archaea are catalytically active. Proc Natl Acad Sci U S A 96:7803–7808. 10393902 10.1073/pnas.96.14.7803 PMC22142

[31] True HL, Celander DW (1998) Protein components contribute to active site architecture for eukaryotic ribonuclease P. J Biol Chem 273:7193–7196. 9516409 10.1074/jbc.273.13.7193

[32] Kikovska E, Svard SG, Kirsebom LA (2007) Eukaryotic RNase P RNA mediates cleavage in the absence of protein. Proc Natl Acad Sci U S A 104:2062–2067. 17284611 10.1073/pnas.0607326104 PMC1892975

[33] Wang MJ, Davis NW, Gegenheimer P (1988) Novel mechanisms for maturation of chloroplast transfer RNA precursors. EMBO J 7:1567–1574. 16453848 10.1002/j.1460-2075.1988.tb02981.x PMC457138

[34] Rossmanith W, Karwan RM (1998) Characterization of human mitochondrial RNase P: novel aspects in tRNA processing. Biochem Biophys Res Commun 247:234–241. 9642109 10.1006/bbrc.1998.8766

[35] Holzmann J, Frank P, Loffler E, Bennett KL, Gerner C, Rossmanith W (2008) RNase P without RNA: identification and functional reconstitution of the human mitochondrial tRNA processing enzyme. Cell 135:462–474. 10.1016/j.cell.2008.09.013 18984158

[36] Gobert A, Gutmann B, Taschner A, Gossringer M, Holzmann J, Hartmann RK, Rossmanith W, Giege P (2010) A single *Arabidopsis* organellar protein has RNase P activity. Nat Struct Mol Biol 17:740–744. 10.1038/nsmb.1812 20473316

[37] Gutmann B, Gobert A, Giege P (2102) PRORP proteins support RNase P activity in both organelles and the nucleus in *Arabidopsis*. Genes Dev 26:1022–1027. 10.1101/gad.189514.112 PMC3360558 22549728

[38] Goldfarb KC, Borah S, Cech TR (2012) RNase P branches out from RNP to protein: organelle-triggered diversification? Genes Dev 26:1005–1009. 10.1101/gad.193581.112 22588715 PMC3360556

[39] Smith D, Pace NR (1993) Multiple magnesium ions in the ribonuclease P reaction mechanism. Biochemistry 32:5273–5281. 8499432 10.1021/bi00071a001

[40] Howard MJ, Lim WH, Fierke CA, Koutmos M (2012) Mitochondrial ribonuclease P structure provides insight into the evolution of catalytic strategies for precursor-tRNA 5' processing. Proc Natl Acad Sci U S A 109:16149–16154. 10.1073/pnas.1209062109 22991464 PMC3479547

[41] Behr MA (2013) Evolution of *Mycobacterium tuberculosis*. Adv Exp Med Biol 783:81–91. 10.1007/978-1-4614-6111-1_4 23468104

[42] Hu YM, Butcher PD, Sole K, Mitchison DA, Coates AR (1998) Protein synthesis is shutdown in dormant *Mycobacterium tuberculosis* and is reversed by oxygen or heat shock. FEMS Microbiol Lett 158:139–145. 9453166 10.1111/j.1574-6968.1998.tb12813.x

